# The Effect of a Comprehensive Life-Style Intervention Program of Diet and Exercise on Four Bone-Derived Proteins, FGF-23, Osteopontin, NGAL and Sclerostin, in Overweight or Obese Children and Adolescents

**DOI:** 10.3390/nu14183772

**Published:** 2022-09-13

**Authors:** Sofia I. Karampatsou, George Paltoglou, Sofia M. Genitsaridi, Penio Kassari, Evangelia Charmandari

**Affiliations:** 1 Division of Endocrinology, Metabolism and Diabetes, First Department of Pediatrics, National and Kapodistrian University of Athens Medical School, ‘Aghia Sophia’ Children’s Hospital, 11527 Athens, Greece; 2 Division of Endocrinology and Metabolism, Center for Clinical, Experimental Surgery and Translational Research, Biomedical Research Foundation of the Academy of Athens, 11527 Athens, Greece

**Keywords:** FGF-23, osteopontin, NGAL, sclerostin, overweight, obesity, bone, childhood, adolescence

## Abstract

The adipose and bone tissues demonstrate considerable interconnected endocrine function. In the present study, we determined the concentrations of fibroblast growth factor-23 (FGF-23), osteopontin, neutrophil gelatinase-associated lipocalin (NGAL) and sclerostin in 345 children and adolescents who were overweight or obese (mean age ± SD mean: 10.36 ± 0.16 years; 172 males, 173 females; 181 prepubertal; and 164 pubertal) before and after their participation in a comprehensive life-style intervention program of diet and exercise for one year. Following the one-year life-style interventions, there was a significant decrease in BMI (*p* < 0.01), FGF-23 (*p* < 0.05), osteopontin (*p* < 0.01) and NGAL (*p* < 0.01), and an increase in sclerostin (*p* < 0.01) concentrations. BMI z-score (b = 0.242, *p* < 0.05) and fat mass (b = 0.431, *p* < 0.05) were the best positive predictors and waist-to-height ratio (WHtR) (b = −0.344, *p* < 0.05) was the best negative predictor of the change of osteopontin. NGAL concentrations correlated positively with HbA1C (b = 0.326, *p* < 0.05), WHtR (b = 0.439, *p* < 0.05) and HOMA-IR (b = 0.401, *p* < 0.05), while BMI (b = 0.264, *p* < 0.05), fat mass (b = 1.207, *p* < 0.05), HDL (b = 0.359, *p* < 0.05) and waist circumference (b = 0.263, *p* < 0.05) were the best positive predictors of NGAL. These results indicate that FGF-23, osteopontin, NGAL and sclerostin are associated with being overweight or obese and are altered in relation to alterations in BMI. They also indicate a crosstalk between adipose tissue and bone tissue and may play a role as potential biomarkers of glucose metabolism. Further studies are required to delineate the physiological mechanisms underlying this association in children and adolescents.

## 1. Introduction

Obesity in childhood and adolescence represents a major public health problem of the last four decades and is associated with increased morbidity and mortality in adulthood. Obesity is characterized by expansion of adipose tissue, either by hypertrophy or by hyperplasia of adipocytes [1]. The main physiologic function of adipose tissue, as an energy reservoir, is complemented by its role in communicating with other tissues and organs, such as the liver, pancreas, heart, muscle and bone, via the release of proteins from the adipocytes, which are called adipokines or adipocytokines [1]. In recent years, several studies have investigated the mechanisms underlying the crosstalk between bone and adipose tissues, given that specific adipokines, such as leptin, adiponectin and resistin, are involved not only in energy homeostasis but also in bone metabolism [2,3]. At the same time, bone-derived factors, such as osteocalcin, are involved in glucose metabolism [2]. It is worth noting that adipocytes and osteoblasts have the same embryologic progenitor, a pluripotential mesenchymal stem cell [2,4].

The fibroblast growth factor (FGF) protein family is implicated in metabolic homeostasis, and specific members of the FGF-19 subfamily, including FGF-19, FGF-21 and FGF-23, have been studied extensively in terms of their endocrine function [5]. FGF-23 is produced by many tissues, although mostly by osteocytes and mature osteoblasts, and acts on the kidneys and parathyroid glands, thereby affecting bone mineralization [5,6]. In order to exert its actions, FGF-23 is activated by the FGF receptor (FGFR) and the co-receptor a-Klotho [6]. Its main and most studied function is the increased catabolism of 1,25(OH)_2_ Vitamin D and the suppression of phosphate reabsorption by the kidney [6]. As a result, elevated concentrations of serum FGF-23 are associated with decreased 1,25(OH)_2_ Vitamin D concentrations and osteomalacia, hypophosphatemia, hyperparathyroidism and chronic kidney disease (CKD). In addition, increased FGF-23 concentrations promote inflammation, erythropoiesis, left ventricular hypertrophy and insulin resistance [5,6]. The main regulators of FGF-23 are 1,25(OH)_2_ Vitamin D, parathormone (PTH) and leptin [5].

Osteopontin was first described approximately 35 years ago as a glycoprotein secreted by bone [7]. It plays an important role in bone metabolism [8] by regulating bone size and density [8,9,10], and increases in osteoporosis [9], knee osteoarthritis [11] and specific progressing cancers, such as osteosarcoma and leukemias [12]. In addition, osteopontin is a pro-fibrotic and pro-inflammatory cytokine that promotes inflammation [13] by stimulating macrophage proliferation [14]. As a result, it contributes to adipogenesis [15] and metabolic-associated fatty liver disease [16], and is upregulated in obesity [14] and insulin resistance [17].

Neutrophil gelatinase-associated lipocalin (NGAL), also known as lipocalin-2, belongs to the lipocalin protein family and is mainly produced by neutrophils [18], but also by the adipose tissue, liver, brain, astrocytes and bone marrow [19]. Another main tissue that secretes NGAL is the bone, and more specifically osteoblasts [20,21]. NGAL is upregulated in metabolic syndrome [22] and in conditions characterized by increased visceral fat mass [23], such as childhood obesity [24].

Sclerostin was first described as a protein produced by the SOST gene and its deficiency results in sclerosteosis, a disorder characterized by extreme bone overgrowth [25]. It is mainly produced by osteocytes and suppresses bone formation [26], but it is also produced by the heart, aorta, liver and kidney [26]. It represents the main inhibitor of the Wnt signaling pathway [27], and administration of its monoclonal antibody results in bone healing after bone fracture [28]. Except for its role in bone, sclerostin also acts as a hormone, interacting with adipose tissue [29]. The increase in sclerostin concentrations could potentially be part of the mechanism where body-weight loss has a negative effect on bone metabolism, resulting in decreased bone mineral density [30,31]. In addition, sclerostin may contribute towards atherosclerosis [32], obesity and insulin resistance [33,34].

The aim of our study was to determine the effect of weight loss on FGF-23, osteopontin, NGAL and sclerostin concentrations in children and adolescents with excess weight and obesity after the implementation of a comprehensive, personalized, life-style intervention program of diet and physical exercise for one year. In addition, we investigated potential associations of these proteins with cardiovascular risk factors and glucose metabolism.

## 2. Materials and Methods

### 2.1. Patients

A total of 345 children and adolescents (mean age ± SD mean age: 10.36 ± 0.16 years; 172 males, 173 females; 181 prepubertal; 164 pubertal) were prospectively recruited to participate in our study for 1 year. The subjects attended our outpatient clinic for the Prevention and Management of Overweight or Obese Children and Adolescents, ‘Aghia Sophia’ Children’s Hospital, Athens, Greece. Subjects were classified as obese (*n* = 220, 63.8%) and overweight (*n* = 125, 36.2%) (Table 1) according to the International Obesity Task Force cutoff points [35]. The study was conducted in accordance with the Declaration of Helsinki and was approved by the Committee on the Ethics of Human Research of ‘Aghia Sophia’ Children’s Hospital (Approval Number: EB-PASCH-MoM: 28 November 2013, Re: 10290-14 May 2013). Written informed consent was obtained in all cases by parents or guardians of the participants, and assent was given by patients older than 7 years.

### 2.2. Methods

All subjects were admitted to the Out-patient Clinic for the Prevention and Management of Overweight or Obese Children and Adolescents, and participated in a personalized, comprehensive, multidisciplinary management life-style intervention program, which has been described in detail previously [36]. Briefly, a single trained observer obtained a detailed medical history and performed the clinical examination. Body weight was measured in light clothing and without shoes using the same scale for all subjects (Seca GmbH & Co. KG., Hamburg, Germany). Standing height was also measured without shoes using a Harpenden’s stadiometer (Holtain Limited, Crymych-Dyfed, UK). Waist and hip circumferences were measured according to WHO STEPS protocol using the same stretch-resistant tape (Seca GmbH & Co. KG., Hamburg, Germany) with the subject in standing position [37]. Systolic (SBP) and diastolic blood pressure (DBP) was determined twice by a sphygmomanometer (Comfort 20/40, Visomat, Parapharm, Metamorphosi, Attiki, Greece) using an appropriate-for-age cuff, and the mean value was calculated. Furthermore, each participant underwent bioelectrical impedance analysis (BIA) (TANITA MC-780U Multi Frequency Segmental Body Composition Analyzer, Amsterdam, The Netherlands) in order to determine fat mass, muscle mass, bone mass, fat-free mass, total body water and basal metabolic rate (BMR). Blood samples for baseline hematologic, biochemical and endocrinologic investigations were obtained at 08:00 h after a 12 h overnight fast. Samples were centrifuged and separated immediately after collection and were stored at −80 °C until assayed.

**Figure 1:**
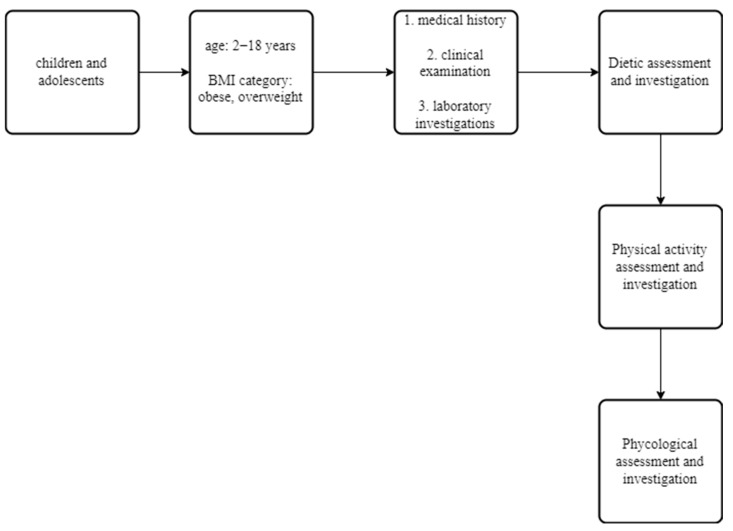
Flowchart of methodology used in the multidisciplinary, life-style intervention program.

All subjects were evaluated by a Pediatrician, Pediatric Endocrinologist, Pediatric Dietician and a professional fitness Personal Trainer, and entered an intervention program that provided personalized advice on diet and physical exercise to patients and their families, as described in detail elsewhere [38]. Subjects with obesity were followed up every month and subjects who were overweight every two months. Upon each follow-up visit, all subjects were evaluated fully and detailed hematologic, biochemical and endocrinologic investigations were performed twelve months later, at the end of the study (Figure 1).

### 2.3. Assessment and Interventions

At initial assessment, all subjects were evaluated by Pediatric Dietitian and a 24-h recall of their diet was performed based on the USDA method [39]. The dietitian recorded the number of meals and snacks, the usual food choices, the person responsible for the preparation of meals, the amount of liquids (water, milk, juices and other beverages) consumed, as well as the frequency and amount of junk food and sweet consumption. Subsequently, children and their parents were informed about the complications of obesity and the importance of a healthier lifestyle for all the family. Also, they were given advices regarding changes in their nutritional habits. They were given recommendations on a healthy diet according to “My Plate” standard, a visualized approach of the USDA 2010 guidelines [40], which included three main meals (breakfast, lunch and dinner) and two snacks (fruits, vegetables) at mid-morning and mid-afternoon. The aim was to recommend a personalized plan for a healthy diet, which would also take into consideration the child’s preferences on food consumption (and therefore not be perceived as boring or difficult), as well as the food availability and preparation while the child is at school or at home.

In addition, a professional fitness Personal Trainer evaluated children and adolescents in relation to their activities and hobbies throughout the week, suggested a personalized exercise program, and encouraged the whole family to avoid a sedentary lifestyle and to follow a physical activity of their choice on a daily basis for 30–45 min, such as walking, jogging, dancing, cycling. The Personal Trainer discussed the child’s interests with the family in order to identify suitable sport activities. The aim was to recommend a personalized physical activity plan, which would not be perceived as compulsory, boring or difficult, but rather as a highly enjoyable and entertaining activity. The assessment of the Personal Trainer was repeated each month and recorded all information about physical activity and exercise.

All subjects included in the study complied with the advice given on diet and exercise, as reported by them and their families. Patients who required psychological or psychiatric input were excluded from the study. At each subsequent appointment, the Pediatrician re-evaluated the anthropometric measurements and the goals set in previous sessions were discussed in detail with the Pediatric Dietitian and the Personal Trainer, as well as the possible difficulties faced by children in achieving their optimal BMI. Detailed hematologic, biochemical and endocrinologic investigations were performed at the beginning and at the end of the study.

### 2.4. Assays

The standard hematologic investigations were determined using the ADVIA 2110i analyzer (Roche Diagnostics, GmbH, Mannheim, Germany). HbA1C was determined using reversed-phase cation exchange High Performance Liquid Chromatography (HPLC) on an automated glycohemoglobin analyzer HA-8160 (Arkray, Kyoto, Japan). Glucose, total cholesterol, triglycerides and high-density lipoprotein cholesterol (HDL) concentrations were determined using the ADVIA 1800 Siemens analyzer (Siemens Healthcare Diagnostics, Tarrytown, NY, USA). Apolipoproteins A1 (Apo-A1), B (Apo-B) and lipoprotein (a) (Lp(a)) concentrations were determined by means of latex particle-enhanced immunonephelometric assays on the BN ProSpec nephelometer (Dade Behring, Siemens Healthcare Diagnostics, Liederbach, Germany).

The concentrations of TSH, T3, free T4, anti-TPO and anti-TG were determined using automated chemiluminescence immunoassays on an IMMULITE 2000 Immunoassay System (Siemens Healthcare Diagnostics Products Ltd., Caernarfon, UK). Insulin and ferritin concentrations were determined using automated electrochemiluminescence immunoassays (ECLIA) (Analyzer Cobas e411, Roche Diagnostics, GmbH, Mannheim, Germany). Total 25-hydroxyvitamin D (25-OH Vitamin D) was determined using an automated electrochemiluminescence immunoassay on the Modular Analytics E170 analyzer.

Adiponectin concentrations were determined using an ELISA kit (Cat. No. BMS2032; eBioscience, ThermoFisher Scientific, Waltham, MA, USA). The sensitivity of the assay was 0.01 ng/mL. The intra-assay CV was 4.2% and the inter-assay CV 3.1%.

FGF-23 concentrations were determined using an ELISA kit (Cat. No. 60-6600; Immutopics, San Clemente, CA, USA). The sensitivity of the assay was 1.5 pg/mL. The intra-assay CV was 3% and the inter-assay CV 6.2%.

Osteopontin concentrations were determined using an ELISA kit (Cat. No. DOST00; R&D Systems, Minneapolis, MN, USA). The sensitivity of the assay was 0.024 ng/mL. The intra-assay CV was 3.1% and the inter-assay CV was 5.9%.

NGAL concentrations were determined using an ELISA kit, (Cat. No. DLCN20; R&D Systems, Minneapolis, MN, USA). The sensitivity of the assay was 0.04 ng/mL. The intra-assay CV was 3.7% and the inter-assay CV was 6.5%.

Sclerostin concentrations were determined using an ELISA kit, (Cat. No. CSB-EL022416HU, Cusabio, Houston, TX, USA). The sensitivity of the assay was 4.68 pg/mL. The intra-assay CV was <10% and the inter-assay CV was <12%.

Insulin resistance (IR) was determined according to the homeostasis model assessment (HOMA) as follows: HOMA-IR = (fasting glucose [mg/dL] × fasting insulin [mU/L])/405. Tri-ponderal mass index (TMI) was calculated using the formula: TMI = mass divided by height cubed. Estimated glomerular filtration rate (eGFR) was calculated using the 2009 bedside Schwartz formula as follows [41]: eGFR = 0.413 × height (cm)/creatinine (mg/dL).

### 2.5. Statistical Analysis

All variables were normally distributed. Results are reported as mean ± SEM. Statistical significance was set at *p* < 0.05. All variables assessed at initial assessment and at 12 months follow-up were compared by employing repeated-measures analysis of variance (ANOVA) tests. Significant main effects were revealed by Fischer’s (LSD) post-hoc test. Correlations of the studied variables were evaluated by the Pearson’s R coefficient. To determine potential predictors of the concentrations and the change of concentrations of FGF23, osteopontin, NGAL and sclerostin, all taken as dependent variables, standard forward, stepwise multiple regression models were employed. In the first model, anthropometric parameters (body weight, height, Body Mass Index (BMI), BMI z-score, TMI, waist and hip circumference, waist-to-hip ratio (WHR) and waist-to-height ratio (WHtR) measurements) at initial assessment were taken as independent variables. In the second model, body composition parameters (fat percentage, fat mass, muscle mass, bone mass, fat free mass, total body water and BMR) at initial assessment were taken as independent variables. In the third model, metabolic syndrome parameters [42] (glucose concentration, SBP, waist circumference, triglycerides and high-density lipoprotein (HDL)] at initial assessment were taken as independent variables. In the fourth model, glucose metabolism and insulin sensitivity parameters (glucose, insulin, HbA1C and HOMA-IR measurements) at initial assessment were taken as independent variables. In the fifth model, adiposity parameters (adiponectin and leptin concentrations, waist circumference, WHtR and fat mass) at initial assessment were taken as independent variables. In the sixth model, bone biochemical parameters (calcium, phosphorus, Alkaline Phosphatase (ALP), PTH, total 25-OH-Vitamin D) at initial assessment were taken as independent variables. All statistical analyses were performed with the Statistica 8 software (StatSoft, Tulsa, OK, USA).

## 3. Results

### 3.1. Clinical Characteristics, and Biochemical, Endocrinologic and Body Composition Parameters of All Subjects at Initial and Annual Assessment

The clinical characteristics of all subjects at baseline are presented in Table 1, Table 2 and Table 3. The study sample consisted of 345 subjects aged 2–18 years (mean age ± SD: 10.36 ± 0.16 years; 172 males, 173 females; 181 prepubertal; 164 pubertal) (Table 1). Children and adolescents with obesity had significantly higher body weight (*p* < 0.01), BMI (*p* < 0.01), BMI z-score (*p* < 0.01) and TMI (*p* < 0.01) than their overweight counterparts. In addition, subjects with obesity had significantly higher waist circumference (*p* < 0.01), hip circumference (*p* < 0.01), WHtR (*p* < 0.01) and DBP (*p* < 0.01) than subjects who were overweight (Table 2).

The clinical characteristics (Table 3A), biochemical parameters (Table 3B), endocrinologic parameters (Table 3C), bone-derived proteins and adipokines (Table 3D), and body composition parameters (Table 3E) of all subjects at initial and annual assessment are presented in Table 3. Following one year of life-style interventions, there was a significant decrease in BMI (*p* < 0.01), BMI z-score (*p* < 0.01) and TMI (*p* < 0.01) in all subjects. More specifically, the percentage of children and adolescents with morbid obesity decreased from 25.8% to 17.4%, while the percentage of subjects with obesity decreased from 38% to 22.9%. The percentage of children and adolescents who were overweight increased from 36.2% to 44.3%, while 15.4% of all subjects had normal BMI at the annual assessment (Table 1). In addition, there was a significant decrease in WHR (*p* < 0.01) and WHtR (*p* < 0.01), and a significant increase in body weight (*p* < 0.01), height (*p* < 0.01), DBP (*p* < 0.01) and hip circumference (*p* < 0.01).

Moreover, following one year of the multidisciplinary management, life-style intervention program, all subjects demonstrated a significant decrease in erythrocyte sedimentation rate (ESR) (*p* < 0.01), hepatic enzymes (aspartate aminotransferase (AST) (*p* < 0.01), alanine transaminase (ALT) (*p* < 0.01), gamma-glutamyl transferase (γGT) (*p* < 0.01)], total cholesterol (*p* < 0.01), low-density lipoprotein (LDL) (*p* < 0.01), and Apo-B (*p* < 0.01) concentrations, and a significant increase in HDL (*p* < 0.01), total 25-OH-vitamin D (*p* < 0.01), PTH (*p* < 0.01) and Lp(a) (*p* < 0.05) concentrations. There was also a significant increase in adiponectin (*p* < 0.01) and sclerostin (*p* < 0.01) concentrations, and a decrease in osteopontin (*p* < 0.01), NGAL (*p* < 0.01), FGF-23 (*p* < 0.05), and leptin (*p* < 0.01) concentrations (Table 3). Although uric acid and Lp(a) concentrations increased, they remained within the reference range of our laboratory (uric acid: 0.0–6.5 mg/dL and Lp(a): <30 mg/dL). Uric acid increases with the progression of puberty [43], and in our study more subjects were pubertal at the annual assessment. Furthermore, although Lp(a) is known to have an inverse relation with BMI, it is mostly genetically determined, with little influence from dietetic and environmental factors [44]; therefore, the decreased BMI at the annual assessment is not likely to have influenced its concentrations.

With respect to the body composition parameters, there was a significant decrease in fat mass percentage (*p* < 0.01) and an increase in muscle mass percentage (*p* < 0.01), bone mass (*p* < 0.01), fat-free mass (*p* < 0.01), total body water (*p* < 0.01) and BMR (*p* < 0.01) (Table 3).

All subjects had normal renal function at initial and annual assessment, as indicated by the urea and creatinine concentrations, and eGFR, which were within the given normal range (Table 3).

### 3.2. Bone-Derived Proteins and Adipokines at Initial and Annual Assessment

Bone-derived proteins and adipokines at initial and annual assessment are presented in Table 4A. Subjects were classified as obese and overweight according to IOTF criteria at initial assessment. Table 4A also presents the respective statistically significant differences between the two groups (as classified at initial assessment) and between initial and annual assessment.

As noted in Table 4A, at initial assessment, leptin concentrations were significantly higher in subjects with obesity compared to subjects who were overweight (*p* < 0.01). At annual assessment, adiponectin concentrations were significantly lower in subjects with obesity compared to subjects with overweight (*p* < 0.05), while Tumor Necrosis Factor-α (TNF-α) (*p* < 0.05) and leptin (*p* < 0.01) concentrations were higher in subjects with obesity compared to subjects who were overweight. In subjects with obesity at initial assessment, there was a significant increase in adiponectin (*p* < 0.05), sclerostin (*p* < 0.01) and TNF-α (*p* < 0.05) concentrations, and a significant decrease in osteopontin (*p* < 0.01), NGAL (*p* < 0.01) and leptin (*p* < 0.01) concentrations compared with annual assessment. In subjects who were overweight at initial assessment, there was a significant increase in adiponectin (*p* < 0.01) and sclerostin (*p* < 0.01) concentrations, and a significant decrease in osteopontin (*p* < 0.01), NGAL (*p* < 0.05), and FGF-23 (*p* < 0.05) concentrations compared with annual assessment (Table 4A).

Table 4B depicts the bone-derived proteins and adipokines at initial and annual assessment in all subjects according to gender (irrespective of pubertal status), while Table 4C,D depicts the same parameters according to gender in patients that were prepubertal (Table 4C) and pubertal (Table 4D) at initial assessment. 

As noted in Table 4B, at initial assessment, adiponectin concentrations were significantly higher in girls compared to boys (*p* < 0.05). At annual assessment, sclerostin (*p* < 0.05) and leptin (*p* < 0.01) concentrations were significantly higher in girls compared to boys. Furthermore, in boys there was a significant increase in adiponectin (*p* < 0.01) and sclerostin (*p* < 0.01) concentrations and a significant decrease in osteopontin (*p* < 0.01), NGAL (*p* < 0.01) and leptin (*p* < 0.01) concentrations at the annual assessment compared with the initial assessment. In girls, there was a significant increase in sclerostin (*p* < 0.01) and a significant decrease in osteopontin (*p* < 0.01), NGAL (*p* < 0.01) and FGF-23 (*p* < 0.05) concentrations at the annual assessment compared with the initial assessment.

As noted in Table 4C, at initial assessment, adiponectin concentrations were significantly higher in girls compared with boys (*p* < 0.05). Furthermore, in pre-pubertal boys, there was a significant increase in adiponectin (*p* < 0.01) and sclerostin (*p* < 0.05) concentrations, and a significant decrease in osteopontin (*p* < 0.01) and NGAL (*p* < 0.05) concentrations at the annual assessment compared with the initial assessment. In pre-pubertal girls, there was a significant increase in sclerostin (*p* < 0.05) and a significant decrease in osteopontin (*p* < 0.05), NGAL (*p* < 0.01) and FGF-23 (*p* < 0.05) concentrations at the annual assessment compared with the initial assessment.

As noted in Table 4D, at initial assessment, adiponectin concentrations were significantly higher in pubertal girls compared to boys (*p* < 0.05). At annual assessment, sclerostin concentrations were higher in girls compared to boys (*p* < 0.05). Furthermore, in pubertal boys there was a significant increase in adiponectin (*p* < 0.01) and sclerostin (*p* < 0.05) concentrations, and a significant decrease in NGAL (*p* < 0.01) concentrations at the annual assessment compared with the initial assessment. In pubertal girls, there was a significant increase in sclerostin (*p* < 0.01), and a significant decrease in osteopontin (*p* < 0.01) and NGAL (*p* < 0.01) concentrations at the annual assessment compared with the initial assessment.

### 3.3. Correlation Coefficient Analysis in Obese, Overweight and All Subjects

The correlation coefficient analysis in obese subjects showed the following: FGF-23 concentrations correlated positively with glucose concentrations (b = 0.151, *p* < 0.05) and sclerostin concentrations (b = 0.643, *p* < 0.05). The change of FGF-23 concentrations correlated positively with TMI (b = 0.174, *p* < 0.05) and fat percentage (b = 0.201, *p* < 0.05) and negatively with calcium (b = −0.171, *p* < 0.05) and sclerostin concentrations (b = −0.269, *p* < 0.05). The osteopontin concentrations correlated positively with glucose (b = 0.403, *p* < 0.05), calcium (b = 0.275, *p* < 0.05), and sclerostin concentrations (b = 0.643, *p* < 0.05) and negatively with the change of FGF-23 (b = −0.280, *p* < 0.05). The change of osteopontin concentrations correlated negatively with glucose concentrations (b = −0.260, *p* < 0.05). NGAL concentrations correlated positively with HbA1C concentrations (b = 0.326, *p* < 0.05). The change of NGAL concentrations correlated negatively with total cholesterol concentrations (b = −0.271, *p* < 0.05) and HbA1C (b = −0.341, *p* < 0.05). Sclerostin concentrations correlated negatively with urea concentrations (b = −0.356, *p* < 0.05). The change of sclerostin concentrations correlated positively with WHtR (b = 0.309, *p* < 0.05) (Supplemental Table S1).

The correlation coefficient analysis in overweight subjects showed the following: FGF-23 concentration correlated negatively with the change of osteopontin (b = −0.263, *p* < 0.05). Osteopontin concentrations correlated positively with PTH (b = 0.260, *p* < 0.05) and vitamin D (b = 0.294, *p* < 0.05) concentrations, and negatively with urea concentrations (b = −0.277, *p* < 0.05), while the change of osteopontin concentrations correlated negatively with vitamin D (b = −0.302, *p* < 0.05) and NGAL (b = −0.398, *p* < 0.05) concentrations. NGAL concentrations correlated positively with WHtR (b = 0.439, *p* < 0.05) and HOMA-IR (b = 0.401, *p* < 0.05). The change of NGAL concentrations correlated negatively with the BMI z-score (b = −0.494, *p* < 0.05). The change of sclerostin concentrations correlated positively with uric acid (b = 0.532, *p* < 0.05), insulin (b = 0.460, *p* < 0.05) and HOMA-IR (b = 0.557, *p* < 0.05), and negatively with waist circumference (b = −0.586, *p* < 0.05), WHR (b = −0.613, *p* < 0.05) and WHtR (b = −0.575, *p* < 0.05) (Supplemental Table S2).

The correlation coefficient analysis of all subjects (obese and overweight) showed the following: FGF-23 concentrations correlated positively with osteopontin concentrations (b = 0.574, *p* < 0.05), sclerostin concentrations (b = 0.443, *p* < 0.05) and the change of sclerostin (b = 0.385, *p* < 0.05), and negatively with the change of osteopontin (b = −0.489, *p* < 0.05). The change of FGF-23 concentrations correlated positively with fat mass (b = 0.313, *p* < 0.05) and the change of osteopontin (b = 0.442, *p* < 0.05), and negatively with osteopontin concentrations (b = −0.534, *p* < 0.05), sclerostin concentrations (b = −0.344, *p* < 0.05) and the change of sclerostin (b = −0.357, *p* < 0.05) (Supplemental Table S3). Osteopontin concentrations correlated positively with glucose concentrations (b = 0.366, *p* < 0.05) and with the change of sclerostin (b = 0.299, *p* < 0.05). The change of osteopontin concentrations correlated negatively with glucose (b = −0.320, *p* < 0.05), NGAL (b = −0.332, *p* < 0.05) and the change of sclerostin (b = −0.352, *p* < 0.05). The change of NGAL concentrations correlated negatively with HbA1C concentrations (b = −0.304, *p* < 0.05). Sclerostin concentrations correlated negatively with urea (b = −0.324, *p* < 0.05) and γGT concentrations (b = −0.329, *p* < 0.05). The change of sclerostin concentration correlated positively with glucose concentrations (b = 0.315, *p* < 0.05) and HOMA-IR (b = 0.300, *p* < 0.05) (Supplemental Table S3).

### 3.4. Multivariate Linear Regression Analysis of Anthropometric Parameters, Body Composition Parameters, Metabolic Syndrome Parameters, Glucose Metabolism Parameters, Adiposity Parameters and Bone Metabolism Parameters

At multivariate linear regression analysis, when anthropometric parameters (weight, height, BMI, BMI z-score, TMI, waist and hip circumference, WHR and WHtR) at initial assessment were taken as independent variables in a standard forward stepwise regression model, BMI z-score (b = 0.242, *p* < 0.05) was the best positive predictor and WHtR (b = −0.344, *p* < 0.05) was the best negative predictor of the change of osteopontin concentrations (dependent variable), BMI (b = 0.264, *p* < 0.05) was the best positive predictor of NGAL(dependent variable), and hip circumference (b = 0.314, *p* < 0.05) was the best positive predictor of sclerostin concentrations (dependent variable).

When body composition parameters (fat percentage, fat mass, muscle mass, bone mass, fat-free mass, total body water and BMR) at initial assessment were taken as independent variables in a standard forward stepwise regression model, fat mass (b = 1.207, *p* < 0.05) was the best positive predictor and BMR was the best negative predictor (b = −1.233, *p* < 0.05) of NGAL concentrations (dependent variable).

When metabolic syndrome parameters at initial assessment (glucose concentration, SBP, waist circumference, triglycerides and HDL) were taken as independent variables in a standard forward stepwise regression model, glucose concentration (b = −0.236, *p* < 0.05) was the best negative predictor of the change of osteopontin concentration (dependent variable), HDL (b = 0.359, *p* < 0.05) and waist circumference (b = 0.263, *p* < 0.05) were the best positive predictors of NGAL concentration (dependent variable), while HDL (b = 0.266, *p* < 0.05) was the best positive predictor of the change of NGAL (dependent variable).

When glucose metabolism parameters (glucose, insulin, HbA1C and HOMA-IR) at initial assessment were taken as independent variables in a standard forward stepwise regression model, glucose concentration was the best positive predictor (b = 0.199, *p* < 0.05) of osteopontin concentrations (dependent variable), glucose was the best negative predictor (b = −0.232, *p* < 0.05) of the change of osteopontin concentrations (dependent variable), and HbA1C was the best negative predictor (b = −0.299, *p* < 0.05) of the change of NGAL concentrations (dependent variable).

When adiposity parameters (adiponectin and leptin concentrations, waist circumference, WHtR and fat mass) at initial assessment were taken as independent variables in a standard forward stepwise regression model, adiponectin concentration (b = 0.202, *p* < 0.05) was the best positive predictor of osteopontin concentrations (dependent variable) and fat mass (b = 0.431, *p* < 0.05) was the best positive predictor of the change of osteopontin (dependent variable).

When bone metabolism parameters (calcium, phosphorus, ALP, PTH and total 25-OH-Vitamin D) at initial assessment were taken as independent variables in a standard forward stepwise regression model, calcium was the best negative predictor of both FGF-23 (b = −0.135, *p* < 0.05) (dependent variable) and the change of FGF23 (b = −0.288, *p* < 0.05) (dependent variable), and calcium (b = 0.175, *p* < 0.05), PTH (b = 0.175, *p* < 0.05) and vitamin D (b = 0.169, *p* < 0.05) concentrations were the best positive predictors of osteopontin concentrations (dependent variable).

## 4. Discussion

In our study, we determined serum FGF-23, osteopontin, NGAL and sclerostin concentrations in children and adolescents who were overweight or obese before and after the implementation of a 1-year personalized, comprehensive, multi-disciplinary, life-style intervention program of diet and physical exercise. We demonstrated that the implementation of this life-style intervention program resulted in a significant decrease in BMI, as well as in FGF-23, osteopontin and NGAL concentrations, and a significant increase in sclerostin concentrations in all subjects. In addition, there was a significant improvement in cardiometabolic risk factors, as indicated by the changes in anthropometric measurements (decrease in DBP, WHR, WHtR, percentage of fat percentage; increase in muscle mass and fat-free mass) and the lipid profile (decrease in total cholesterol, LDL, ApoB; increase in HDL concentrations). To the best of our knowledge, this is the first study in children and adolescents with normal renal function that demonstrates the association of FGF-23, osteopontin, NGAL and sclerostin concentrations with obesity, cardiovascular risk factors and glucose homeostasis following a 1-year life-style intervention program.

FGF-23 is produced mostly in the bone, it regulates phosphorus and vitamin D homeostasis [6], and interacts with other tissues, such as the adipose tissue [5,6]. In our study, we demonstrated that FGF-23 concentrations decreased along with the decrease in BMI, while the change in FGF-23 concentrations correlated positively with TMI in obese subjects and fat mass in all subjects. Our findings concur with several studies performed in adults and adolescents without chronic kidney diseases (CKD), which demonstrated that FGF-23 was significantly higher in overweight and obese subjects (especially those with visceral obesity), and correlated positively with markers of obesity (BMI, WC, WHR, visceral fat mass and subcutaneous fat area) [45,46,47,48]. Furthermore, FGF-23 was higher in adults with normal BMI and abdominal obesity, indicating an increased metabolic and cardiovascular risk [49], as well as in adolescents with obesity and those with cardiac hypertrophy, suggesting that it may be an early marker of cardiac injury in adolescents with obesity [50]. FGF-23 participates in the communication of adipose tissue with the bone, probably through leptin, which stimulates FGF-23 expression via the JAK-STAT3 pathway [51].

FGF-23 also plays a major role in glucose homeostasis. Bar et al. demonstrated that insulin can directly down-regulate the trascription of the FGF-23 gene through FOXO1 [52]. In our study, we demonstrated that FGF-23 concentrations correlated positively with glucose concentrations in obese subjects. These findings are also in agreement with previous studies that demonstrated that FGF-23 concentrations were higher in children and adolescents with impaired glucose tolerance compared to a control group [53], and correlated positively with insulin and HOMA score [50]. Moreover, in a study of 1104 adults, FGF-23 concentrations correlated positively with HOMA-IR [54]. Our results, along with those of the above studies, indicate that FGF-23 could be a biomarker of insulin resistance.

Osteopontin is a protein secreted by osteoblasts and osteoclasts [7], connecting bone with other tissues, including the adipose tissue [14]. Osteopontin, as an inflammatory cytokine, communicates with the adipose tissue and promotes the inflammation that takes place in adipocytes during obesity, although the exact mechanism is not completely understood. In our study, we showed that osteopontin concentrations decreased along with the decrease in BMI. In addition, BMI z-score was the best positive predictor of the change of osteopontin, while WHtR was the best negative predictor of the change of osteopontin concentrations. Previous studies also showed that osteopontin was significantly higher in adolescents who were overweight or obese than a control group of normal BMI [55], and correlated positively with cardiovascular risk factors in children with obesity [56]. Interestingly, osteopontin concentrations are significantly higher in children [57], and urine osteopontin concentrations are increased in children with obesity compared with lean children [24].

We also demonstrated that fat mass was the best positive predictor of the change of osteopontin, while adiponectin concentrations were the best positive predictor of the osteopontin concentrations, indicating an association between osteopontin and fat mass. Osteopontin is expressed not only in the bone but also in the adipose tissue, especially in obese subjects, and as a result it could act in the bone as an endocrine, paracrine and autocrine factor [58,59]. Qian et al., showed that osteopontin and adiponectin correlated positively in adults with rheumatoid arthritis [60], while Kurata et al. showed that adiponectin was an independent predictor of osteopontin in adults with essential hypertension, although the correlation was negative [61]. Moreover, in studies in obese and non-obese adults, osteopontin correlated positively with body fat [59,62].

Several studies have demonstrated the role of osteopontin in glucose homeostasis [17]. The underlying mechanism may relate to the fact that osteopontin promotes hepatic endoplasmic reticulum stress through NK cells, which leads to insulin resistance and hepatic steatosis [17]. In our study, osteopontin concentrations correlated positively with glucose concentrations, and glucose concentrations were the best positive predictor of osteopontin concentrations and the best negative predictor of the change of osteopontin concentrations. In a study in high-fed diet mice, when osteopontin expression was neutralized by antibodies, insulin resistance was reduced, suggesting that osteopontin may be a potential pharmaceutical target for cardiometabolic disorders in subjects with obesity [63]. Moreover, osteopontin deficiency improved hepatic steatosis, insulin sensitivity and glucose homeostasis [64]. Similar to our findings, Hamilcikan et al. demonstrated that in children and adolescents with obesity, increased osteopontin concentrations were an important risk factor for insulin resistance [65], while in adults, osteopontin correlated positively with fasting glucose and insulin [66]. Intrestingly, osteopontin could increase before the onset of diabetes, and as a result could be a potential biomarker of impaired glucose tolerance, before diabetes appears [66].

NGAL is a protein produced mainly by neutrophils, but also by bone, and is involved in metabolism [19,20]. It is up-regulated in obesity and promotes inflammation through inflammatory cytokines IL-1β and TNF-α [67]. Our results reveal that NGAL concentrations decrease along with the decrease in BMI following the implementation of a life-style intervention program, while the change of NGAL concentrations correlated negatively with BMI z-score only in overweight subjects. In addition, BMI was the best positive predictor of NGAL. Our results are in accordance with previous studies in adults, which showed that NGAL concentrations correlated positively with body weight and BMI [22,68]. Moreover, urine NGAL is increased in children with obesity compared with their lean counterparts [24,69]. In a study of obese children, no correlation was found in NGAL concentrations, when compared to healthy children, however, the sample size was small [70]. Interestingly, children with obesity carrying a specific mutation of the NGAL gene could easier reduce their BMI-z-score after an 8-week interventional program [71].

Further to the above, we showed that fat mass was the best positive predictor and BMR was the best negative predictor of NGAL concentrations. Our results concur with those of other studies in adults demonstrating that NGAL concentrations were increased in subjects with visceral obesity [45] and had a positive correlation with fat mass, regardless of their BMI [23,45]. Interestingly, in prepubertal children, urinary NGAL correlated with the percentage of body fat mass [69]. To the best of our knowledge, ours is the first study that correlated NGAL with BMR.

NGAL also plays an important role in metabolic syndrome and glucose homeostasis [22], regulating insulin secretion by the pancreatic beta cells [20]. More specifically, it is speculated that NGAL could influence the fuction of beta cells and insulin sensitivity via iron metabolism, since elevated iron and ferritin concentrations can promote pancreatic injury and insulin resistance [72]. In our study, NGAL concentrations correlated positively with HOMA-IR and HbA1C, while the change of NGAL concentrations correlated negatively with HbA1C. Furthermore, HbA1C at initial assessment was the best negative predictor of the change of NGAL concentrations. Once again, our findings concur with those of previous studies showing that increased NGAL concentrations correlate positively with metabolic risk [22], plasma glucose and HbA1C [68,72,73]. Urinary NGAL is increased in children with obesity and insulin resistance, probably due to tubular kidney damage prior to the development of diabetes mellitus [24,74], and correlates positively with HbA1C in adolescents and adults with diabetes mellitus type I [75]. The above results indicate that NGAL might be a biomarker of insulin resistance, although more studies are needed to explore the underlying mechanism and its relation to the pathogenesis of insulin resistance.

Sclerostin is another bone-derived protein [29] that suppreses bone formation [26]. In addition, it interacts with adipose tissue and influences metabolic homeostasis [34]. In the present study, sclerostin concentrations increased along with the decrease in BMI, despite the increase in bone mass. This may occur because sclerostin participates in a negative feedback mechanism of bone remodeling, and the increase in bone mass may precede the increase in sclerostin [26]. As for the decrease in BMI, our findings are in agreement with previous studies, which showed that in adult women who underwent Roux-en-Y gastric bypass or laparoscopic sleeve gastrectomy, sclerostin concentrations increased 1 and 6 months after the intervention [30]. Similar results were noted in adults who participated in an intervention program of diet [31], where sclerostin concentrations correlated negatively with BMI [76]. Similarly, in children with obesity, sclerostin concentrations were decreased compared with the control group [77], and correlated negatively with BMI [78]. More studies are required to clarify the role of sclerostin in the pathogenesis of obesity.

Finally, we demonstrated that sclerostin concentrations were higher in pubertal girls compared with pubertal boys. Similar results were documented in a study of adults with T1DM, where women demonstrated higher concentrations of sclerostin compared with men [79], and in a population of athletes and non-athletes [80]. The higher concentrations of sclerostin in female subjects could be due to the positive correlation of sclerostin expression with estrogens [81], which increase after the onset of puberty.

Our study has several strengths. The main strength of our study is that it is the first one to determine these bone-derived proteins in children and adolescents with normal renal function. It is also the first study that demonstrates the association of FGF-23, osteopontin, NGAL and sclerostin concentrations with obesity, cardiovascular risk factors, glucose homeostasis and BMR following a 1-year life-style intervention program. Furthermore, the sample size of children and adolescents is large. The only limitation is, perhaps, that we did not include a control group of children and adolescents of normal BMI. However, this was not necessary according to the study design, because our aim was to determine the alterations of these four bone-derived proteins in children and adolescents with obesity and overweight following reduction in their BMI.

## 5. Conclusions

In conclusion, a personalized, multi-disciplinary, life-style intervention program of diet and exercise can be effective in decreasing the BMI and improving cardiovascular risk factors, thereby addressing the epidemic of childhood obesity. More importantly, the decrease in BMI was associated with a decrease in FGF-23, osteopontin and NGAL concentrations, and an increase in sclerostin concentrations. In addition, the bone-derived proteins correlated with anthropometric measurements and indices of glucose, lipid and bone metabolism. These results suggest a crosstalk between adipose tissue and the bone, as endocrine organs, and indicate that FGF-23, osteopontin and NGAL may serve as biomarkers of obesity and its pathophysiologic implications, such as insulin resistance. Further studies are required to delineate the physiological mechanisms underlying this association in children and adolescents.

## Figures and Tables

**Table 1:** Gender, pubertal and BMI category of all subjects at initial and annual assessment.

	Initial Assessment	Annual Assessment
Gender		
Male	172 (49.9%)	
Female	173 (50.1%)	
Pubertal status		
Prepubertal	181 (52.5%)	115 (33.3%)
Pubertal	164 (47.5%)	230 (66.7%)
BMI category		
Morbidly obese	89 (25.8%)	60 (17.4%)
Obese	131 (38%)	79 (22.9%)
Overweight	125 (36.2%)	153 (44.3%)
Normal BMI	-	53 (15.4%)

Abbreviations: BMI, body mass index; categorical variables are presented as frequencies (percentages).

**Table 2:** Clinical characteristics of all subjects at initial assessment.

	Obese (*n* = 220)	Overweight (*n* = 125)	*p*
Age (years)	10.26 ± 0.21	10.53 ± 0.24	NS
Height (cm)	145.24 ± 1.30	145.56 ± 1.41	NS
BW (kg)	64.53 ± 1.68	52.82 ± 1.38	**<0.01**
BMI (kg/m^2^)	28.71 ± 0.32	24.25 ± 0.20	**<0.01**
BMI z-score	3.17 ± 0.07	1.55 ± 0.02	**<0.01**
TMI (kg/m^3^)	19.91 ± 0.23	16.74 ± 0.10	**<0.01**
Waist circumference (cm)	87.58 ± 1.05	78.03 ± 1.03	**<0.01**
Hip circumference (cm)	93.20 ± 1.14	84.98 ± 1.09	**<0.01**
WHR	0.95 ± 0.01	0.92 ± 0.01	NS
WHtR	0.60 ± 0.01	0.54 ± 0.01	**<0.01**
SBP (mmHg)	112.67 ± 1.33	109.02 ± 1.05	NS
DBP (mmHg)	65.35 ± 1.08	60.76 ± 0.97	**<0.01**

Abbreviations: BMI, body mass index; BW, body weight; DBP, diastolic blood pressure; SBP, systolic blood pressure; TMI, tri-ponderal mass index; WHR, waist-to-hip ratio; WHtR, waist-to-height ratio; All variables are presented as mean ± SEM; Subjects were classified as obese and overweight according to IOTF criteria at initial assessment. The Table presents comparisons among the two groups; All measured variables were compared by employing one-way ANOVA; Significant main effects were revealed by the LSD post-hoc test; Statistical significance was set at *p* < 0.05, while strong significance at *p* < 0.01 is also noted; NS: nonsignificant (*p* > 0.05) difference; Statistically significant associations are shown in bold.

**Table 3:** Characteristics of all subjects at initial and annual assessment. (**A**) Clinical characteristics of all subjects. (**B**) Biochemical parameters. (**C**) Endocrinologic parameters. (**D**) Bone-derived proteins and adipokines. (**E**) Body composition parameters.

(A)			
	Initial Assessment	Annual Assessment	*p*-Value
Age (years)	10.36 ± 0.16	11.46 ± 0.16	**<0.01**
BW (kg)	60.27 ± 1.22	62.18 ± 1.18	**<0.01**
Height (cm)	145.36 ± 0.97	151.43 ± 0.98	**<0.01**
BMI (kg/m^2^)	27.09 ± 0.24	25.84 ± 0.24	**<0.01**
BMI z-score	2.58 ± 0.06	2.02 ± 0.06	**<0.01**
TMI (kg/m^2^)	18.76 ± 0.17	17.24 ± 0.21	**<0.01**
SBP (mmHg)	111.29 ± 0.92	113.10 ± 0.88	NS
DBP (mmHg)	63.62 ± 0.78	67.88 ± 0.75	**<0.01**
Waist circumference (cm)	84.34 ± 0.82	85.51 ± 1.17	NS
Hip circumference (cm)	90.44 ± 0.87	92.94 ± 1.10	**<0.01**
WHR	0.94 ± 0.01	0.92 ± 0.01	**<0.01**
WHtR	0.58 ± 0.01	0.56 ± 0.01	**<0.01**
**(B)**			
	**Initial Assessment**	**Annual Assessment**	***p*** **-Value**
ESR (mm/hr)	19.64 ± 0.77	16.36 ± 0.67	**<0.01**
Ferritin (μg/L)	59.07 ± 1.77	58.29 ± 2.20	NS
Folic acid (ng/mL)	12.88 ± 0.51	11.04 ± 0.33	**<0.01**
Iron (μg/dL)	85.61 ± 2.07	79.36 ± 6.34	NS
Glucose (mg/dL)	78.69 ± 0.48	82.80 ± 2.54	NS
Urea (mg/dL)	29.17 ± 0.34	27.87 ± 0.34	**<0.01**
Creatinine (mg/dL)	0.52 ± 0.05	0.53 ± 0.01	NS
eGFR (mL/min/1.73 m^2^)	132.07 ± 1.50	123.51 ± 1.42	**<0.01**
AST (U/L)	24.22 ± 0.34	22.25 ± 0.36	**<0.01**
ALT (U/L)	22.80 ± 0.88	19.70 ± 0.63	**<0.01**
γGT (U/L)	15.04 ± 0.31	13.84 ± 0.30	**<0.01**
ALP (U/L)	234.45 ± 4.19	213.77 ± 4.46	**<0.01**
Phosphorus (mg/dL)	4.80 ± 0.05	4.67 ± 0.02	**<0.01**
Albumin (g/dL)	4.65 ± 0.02	4.66 ± 0.02	NS
Cholesterol (mg/dL)	159.67 ± 1.62	152.60 ± 2.10	**<0.01**
Triglycerides (mg/dL)	82.90 ± 2.38	80.90 ± 2.55	NS
HDL (mg/dL)	50.08 ± 0.68	53.41 ± 0.75	**<0.01**
LDL (mg/dL)	94.35 ± 1.28	88.28 ± 1.30	**<0.01**
Uric acid (mg/dL)	4.89 ± 0.06	6.19 ± 0.56	**<0.05**
K (mmol/L)	4.39 ± 0.02	4.40 ± 0.02	NS
Na (mmol/L)	140.73 ± 0.08	140.38 ± 0.13	NS
Cl (mmol/L)	102.66 ± 0.16	101.10 ± 0.83	NS
Ca (mmol/L)	9.95 ± 0.02	9.81 ± 0.02	**<0.01**
Apo-A1 (mg/dL)	141.67 ± 1.30	141.83 ± 1.23	NS
Apo-B (mg/dL)	76.09 ± 1.07	71.77 ± 0.93	**<0.01**
Lp(a) (mg/dL)	16.74 ± 1.31	17.93 ± 1.49	**<0.05**
**(C)**			
	**Initial Assessment**	**Annual Assessment**	***p*** **-Value**
TSH (μUI/mL)	2.97 ± 0.08	2.91 ± 0.09	NS
FT4 (mg/dL)	1.15 ± 0.03	1.12 ± 0.01	NS
T3 (ng/dL)	147.63 ± 1.43	141.07 ± 1.56	**<0.01**
Anti-TG (IU/mL)	20.80 ± 0.40	23.64 ± 1.98	NS
Anti-TPO (IU/mL)	15.78 ± 1.45	16.39 ± 2.18	NS
IGF-1 (ng/mL)	307.27 ± 9.25	389.39 ± 10.89	**<0.01**
IGFBP-3 (μg/mL)	5.14 ± 0.06	5.47 ± 0.07	NS
Androstenedione (ng/mL)	1.07 ± 0.08	2.44 ± 1.11	NS
Testosterone (ng/mL)	56.36 ± 4.79	89.56 ± 6.91	**<0.01**
DHEA-s (μg/dl)	113.26 ± 4.92	139.97 ± 5.76	**<0.01**
Prolactin (ng/mL)	11.75 ± 0.39	12.18 ± 0.38	**<0.05**
LH (mUI/mL)	2.30 ± 0.32	2.96 ± 0.19	**<0.01**
FSH (mUI/mL)	5.22 ± 2.54	3.32 ± 0.14	NS
E2 (pg/mL)	14.79 ± 1.67	17.47 ± 1.25	**<0.01**
PTH (pg/mL)	33.84 ± 0.70	36.93 ± 0.68	**<0.01**
Vitamin D (ng/mL)	22.79 ± 0.54	24.52 ± 0.52	**<0.01**
ACTH (pg/mL)	34.09 ± 3.96	27.94 ± 1.08	NS
Cortisol (μg/dL)	15.85 ± 0.44	13.81 ± 0.34	**<0.01**
Insulin (μUI/mL)	16.18 ± 0.54	15.80 ± 0.56	NS
SHBG (nmol/L)	45.72 ± 1.79	46.79 ± 1.64	NS
HbA1c (%)	7.01 ± 1.59	5.27 ± 0.01	NS
HOMA-IR	3.13 ± 0.11	3.15 ± 0.13	NS
**(D)**			
	**Initial Assessment**	**Annual Assessment**	***p*** **-Value**
Adiponectin (pg/mL)	21.61 ± 0.95	25.91 ± 1.07	**<0.01**
Osteopontin (ng/mL)	30.63 ± 1.80	21.22 ± 1.15	**<0.01**
NGAL (ng/mL)	19.97 ± 1.41	10.76 ± 0.77	**<0.01**
Sclerostin (pg/mL)	2.00 ± 0.39	4.82 ± 0.59	**<0.01**
FGF-23 (pg/mL)	10.38 ± 2.63	7.50 ± 1.76	**<0.05**
Leptin (ng/mL)	29.79 ± 1.32	23.94 ± 0.99	**<0.01**
TNF-α (pg/mL)	3.80 ± 0.12	3.99 ± 0.09	NS
**(E)**			
	**Initial Assessment**	**Annual Assessment**	***p*** **-Value**
Fat Percentage (%)	35.03 ± 0.35	32.85 ± 0.33	**<0.01**
Fat Mass (kg)	21.48 ± 0.60	20.63 ± 0.54	NS
Muscle Mass Percentage (%)	36.65 ± 0.74	38.68 ± 0.66	**<0.01**
Bone Mass (kg)	1.99 ± 0.04	2.17 ± 0.08	**<0.01**
Fat-Free Mass (kg)	38.65 ± 0.78	40.75 ± 0.70	**<0.01**
Total Body Water	28.29 ± 0.57	29.88 ± 0.51	**<0.01**
BMR (Kilojoule)	6428.30 ± 86.51	6585.94 ± 77.27	**<0.01**

Abbreviations: ACTH, adrenocorticotropic hormone; ALP, alkaline phosphatase; ALT, alanine transaminase; anti-TG, antibodies against thyroglobulin; anti-TPO, thyroid peroxidase antibodies; apo-A1, apolipoprotein A1; apo-B, apolipoprotein B; AST, aspartate aminotransferase; BMI, body mass index; BMR, basal metabolic rate; BW, body weight; DBP, diastolic blood pressure; DHEA-s, dehydroepiandrosterone sulfate; E2, estradiol; eGFR, estimated glomerular filtration rate; ESR, erythrocyte sedimentation rate; FGF-23, fibroblast growth factor-23; FSH, follicle stimulating hormone; FT4, free thyroxine; γGT, gamma-glutamyl transferase; HbA1C, hemoglobin A1C; HDL, high density lipoprotein; HOMA-IR, homeostatic model assessment for insulin resistance; IGF-1, insulin-like growth factor 1; IGFBP-3: IGF-binding protein 3; LDL, low density lipoprotein; LH, luteinizing hormone; Lp(a), lipoprotein a; NGAL, neutrophil gelatinase associated lipocalin; PTH, parathormone; SBP, systolic blood pressure; SHBG, sex hormone-binding globulin; T3, triiodothyronine; TMI, tri-ponderal mass index; TNF-α, tumor necrosis factor-a; TSH, thyroid stimulating hormone; vitamin D, total 25-OH-vitamin D; WHR, waist-to-hip ratio; WHtR, waist-to-height ratio; all variables are presented as mean ± SE of mean; all measured variables were compared by employing one-way ANOVA; significant main effects were revealed by the LSD post-hoc test; statistical significance was set at *p* < 0.05, while strong significance at *p* < 0.01 is also noted; NS: nonsignificant (*p* > 0.05) difference; statistically significant associations are shown in bold.

**Table 4:** (**A**) Bone-derived proteins and adipokines at initial and annual assessment. (**B**) Bone-derived proteins and adipokines at initial and annual assessment: Comparisons according to gender (irrespective of pubertal status). (**C**) Bone-derived proteins and adipokines at initial and annual assessment: Comparisons by gender of overweight and obese subjects, who were pre-pubertal at initial assessment. (**D**) Bone-derived proteins and adipokines at initial and annual assessment: Comparisons by gender of overweight and obese subjects, who were pubertal at initial assessment.

(A)							
	Initial Assessment			Annual Assessment			
	Obese	Overweight	*p*_within_	Obese	Overweight	*p*_within_	*p*_between timepoints_ (obese/overweight)
Adiponectin (pg/mL)	20.77 ± 1.16	22.85 ± 1.58	NS	24.07 ± 1.23	29.29 ± 1.89	**0.05**	**0.05**/**0.01**
Osteopontin (ng/mL)	30.91 ± 2.24	30.21 ± 2.93	NS	21.52 ± 1.49	20.42 ± 1.69	NS	**0.01**/**0.01**
NGAL (ng/mL)	21.16 ± 1.83	16.62 ± 1.82	NS	10.68 ± 0.86	10.76 ± 1.52	NS	**0.01**/**0.05**
Sclerostin (pg/mL)	2.06 ± 0.49	1.75 ± 0.55	NS	5.01 ± 0.68	4.08 ± 1.05	NS	**0.01**/**0.01**
TNF-α (pg/mL)	3.67 ± 0.15	4.04 ± 0.17	NS	4.05 ± 0.10	3.90 ± 0.18	**0.05**	**0.05**/NS
FGF-23 (pg/mL)	9.44 ± 2.73	11.92 ± 5.25	NS	7.63 ± 2.36	7.30 ± 2.52	NS	NS/**0.05**
Leptin (ng/mL)	32.62 ± 1.71	24.33 ± 1.86	**0.01**	24.96 ± 1.21	21.84 ± 1.61	**0.05**	**0.01**/NS
**(** **B)**							
	**Initial Assessment**			**Annual Assessment**			
	**Boys**	**Girls**	***p*** **_within_**	**Boys**	**Girls**	***p*** **_within_**	***p*** **_between timepoints_** **(Boys/Girls)**
Adiponectin (pg/mL)	18.71 ± 1.14	24.48 ± 1.49	**0.05**	24.97 ± 1.50	26.87 ± 1.51	NS	**0.01**/NS
Osteopontin (ng/mL)	31.76 ± 2.51	29.58 ± 2.59	NS	23.18 ± 1.83	19.38 ± 1.40	NS	**0.01**/**0.01**
NGAL (ng/mL)	18.61 ± 1.97	21.31 ± 2.02	NS	9.93 ± 1.04	11.58 ± 1.13	NS	**0.01**/**0.01**
Sclerostin (pg/mL)	1.68 ± 0.62	2.32 ± 0.49	NS	3.80 ± 0.83	5.82 ± 0.80	**<0.05**	**0.01**/**0.01**
TNF-α (pg/mL)	3.88 ± 0.20	3.72 ± 0.12	NS	4.02 ± 0.14	3.96 ± 0.13	NS	NS/NS
FGF-23 (pg/mL)	8.16 ± 2.29	12.56 ± 4.70	NS	6.31 ± 1.69	8.66 ± 3.07	NS	NS/**0.05**
Leptin (ng/mL)	27.88 ± 1.85	31.72 ± 1.86	NS	20.19 ± 1.33	27.73 ± 1.41	**0.01**	**0.01**/NS
**(C)**							
	**Initial Assessment**			**Annual Assessment**			
	**Boys**	**Girls**	***p*** **_within_**	**Boys**	**Girls**	***p*** **_within_**	***p*** **_between timepoints_** **(Boys/Girls)**
Adiponectin (pg/mL)	21.34 ± 1.71	27.23 ± 2.24	0.05	27.94 ± 2.03	29.01 ± 2.12	NS	**0.01**/NS
Osteopontin (ng/mL)	33.99 ± 3.90	29.60 ± 3.90	NS	23.25 ± 2.66	21.21 ± 2.06	NS	**0.01**/**0.05**
NGAL (ng/mL)	17.90 ± 2.31	22.26 ± 2.75	NS	10.84 ± 1.89	9.91 ± 1.39	NS	**0.05**/**0.01**
Sclerostin (pg/mL)	0.89 ± 0.39	2.57 ± 1.07	NS	2.88 ± 1.03	4.95 ± 1.37	NS	**0.05**/**0.05**
TNF-α (pg/mL)	3.87 ± 0.21	3.69 ± 0.15	NS	4.07 ± 0.21	4.07 ± 0.17	NS	NS/NS
FGF-23 (pg/mL)	9.93 ± 3.50	13.21 ± 8.08	NS	6.98 ± 2.67	7.36 ± 3.75	NS	NS/**0.05**
Leptin (ng/mL)	26.12 ± 2.68	26.62 ± 2.30	NS	20.90 ± 1.61	23.90 ± 1.62	NS	NS/NS
**(D)**							
	**Initial Assessment**			**Annual Assessment**			
	**Boys**	**Girls**	***p*** **_within_**	**Boys**	**Girls**	***p*** **_within_**	***p*** **_between timepoints_** **(Boys/Girls)**
Adiponectin (pg/mL)	15.55 ± 1.35	21.41 ± 1.87	**0.05**	21.31 ± 2.16	24.65 ± 2.15	NS	**0.01**/NS
Osteopontin (ng/mL)	29.48 ± 3.16	29.56 ± 3.45	NS	23.12 ± 2.55	17.59 ± 1.88	NS	NS/**0.01**
NGAL (ng/mL)	19.34 ± 3.26	20.66 ± 2.86	NS	8.99 ± 0.81	12.70 ± 1.62	NS	**0.01**/**0.01**
Sclerostin (pg/mL)	2.38 ± 1.11	2.17 ± 0.48	NS	4.62 ± 1.27	6.33 ± 0.99	**0.05**	**0.05**/**0.01**
TNF-α (pg/mL)	3.90 ± 0.37	3.74 ± 0.19	NS	3.97 ± 0.17	3.85 ± 0.19	NS	NS/NS
FGF-23 (pg/mL)	6.42 ± 2.99	11.92 ± 4.98	NS	5.61 ± 2.07	9.92 ± 4.86	NS	NS/NS
Leptin (ng/mL)	29.99 ± 2.51	37.08 ± 2.85	**0.05**	19.33 ± 2.21	31.70 ± 2.24	**0.01**	NS/NS

Subjects were classified as obese or overweight according to IOTF criteria at initial assessment. The respective statistically significant differences between the two groups (as classified at initial assessment) and between initial and annual assessment are also presented. Abbreviations: FGF-23, fibroblast growth factor-23; neutrophil gelatinase associated lipocalin; TNF-α, tumor necrosis factor-a; all variables are presented as mean ± SE of mean; all measured variables were compared by employing one-way ANOVA; significant main effects were revealed by the LSD post-hoc test; statistical significance was set at *p* < 0.05, while strong significance at *p* < 0.01 is also noted; NS: nonsignificant (*p* > 0.05) difference. Statistically significant associations are shown in bold.

## Data Availability

The data presented in this study are available on request from the corresponding author. The data are not publicly available due to privacy restrictions.

## Supplementary Materials

The following supporting information can be downloaded at: https://www.mdpi.com/article/10.3390/nu14183772/s1, Table S1: Correlation coefficient of the assessed variables of obese subjects at initial assessment, Table S2: Correlation coefficient of the assessed variables of overweight subjects at initial assessment, Table S3: Correlation coefficient of the assessed variables of all subjects at initial assessment (overweight and obese).
