# Supplementary material for: The Effect of a Comprehensive Life-Style Intervention Program of Diet and Exercise on Four Bone-Derived Proteins, FGF-23, Osteopontin, NGAL and Sclerostin, in Overweight or Obese Children and Adolescents
Source: Nutrients. 2022 Sep 13;14(18):3772. doi: 10.3390/nu14183772 (PMC9505283; doi:10.3390/nu14183772)
Supplement: Supplementary file 1 [file nutrients-14-03772-s001.zip › nutrients-1876089-supplementary.pdf]

Supplemental Table S1. Correlation coefficient of the assessed variables of obese subjects at initial assessment

	FGF-23_t0	Change of FGF-23	Osteopontin_t0	Change of Osteopontin	NGAL_t0	Change of NGAL	Sclerostin_t0	Change of Sclerostin
**BW_t0**	b= 0.005, p>0.05	b= 0.058, p>0.05	b= -0.068, p>0.05	b= 0.034, p>0.05	b= 0.135, p>0.05	b= 0.020, p>0.05	b=0.121, p>0.05	b=0.079, p>0.05
**BMI_z-score_t0**	b= -0.040, p>0.05	b=0.147, p>0.05	b= -0.146, p>0.05	b=0.090, p>0.05	b=-0.040, p>0.05	b= 0.125, p>0.05	b= 0.098, p>0.05	b=0.097, p>0.05
**TMI_t0**	b= -0.062, p>0.05	**b=0.174, p<0.05**	b=-0.132, p>0.05	b=0.065, p>0.05	b= 0.036, p>0.05	b=0.107, p>0.05	b= 0.107, p>0.05	b=0.166, p>0.05
**Waist circumferance_t0**	b= 0.015, p>0.05	b=0.040, p>0.05	b= 0.043, p>0.05	b=-0.130, p>0.05	b=0.100, p>0.05	b=0.595, p>0.05	b= 0.048, p>0.05	b=0.210, p>0.05
**WHR_t0**	b= -0.017, p>0.05	b=0.0004, p>0.05	b= -0.119, p>0.05	b=0.079, p>0.05	b= 0.029, p>0.05	b=0.059, p>0.05	b= -0.178, p>0.05	b=-0.064, p>0.05
**WHtR_t0**	b= -0.016, p>0.05	b=0.081, p>0.05	b= 0.031, p>0.05	b=-0.168, p>0.05	b= 0.021, p>0.05	b=0.086, p>0.05	b= -0.042, p>0.05	**b=0.309, p<0.05**
**Glucose_t0**	**b=0.151, p<0.05**	b=0.149, p>0.05	**b=0.403, p<0.05**	**b=-0.260, p<0.05**	b=-0.004, p>0.05	b=-0.013, p>0.05	b=0.216, p>0.05	b=0.114, p>0.05
**Insulin_t0**	b=-0.001, p>0.05	b=0.017, p>0.05	b=0.010, p>0.05	b=-0.050, p>0.05	b=-0.020, p>0.05	b=0.087, p>0.05	b=0.021, p>0.05	b=0.227, p>0.05
**HbA1c_t0**	b=-0.014, p>0.05	b=0.011, p>0.05	b=-0.035, p>0.05	b=0.022, p>0.05	**b=0.326, p<0.05**	**b=-0.341, p<0.05**	b=-0.057, p<0.05	b=0.015, p<0.05
**HOMA-IR_t0**	b=0.022, p>0.05	b=-0.027, p<0.05	b=0.068, p>0.05	b=-0.075, p<0.05	b=-0.014, p>0.05	b=0.084, p<0.05	b=0.061, p>0.05	b=0.232, p<0.05
**Urea_t0**	b=0.035, p>0.05	b=0.020, p>0.05	b=0.069, p>0.05	b=-0.130, p>0.05	b=-0.100, p>0.05	b=0.082, p>0.05	**b=-0.356, p<0.05**	b=0.102, p>0.05
**Creatinine_t0**	b=-0.054, p>0.05	b=0.051, p>0.05	b= 0.0009, p>0.05	b=-0.026, p>0.05	b=0.018, p>0.05	b=0.042, p>0.05	b=-0.165, p>0.05	b=-0.015, p>0.05
**eGFR_t0**	b=-0.078, p>0.05	b=-0.061, p>0.05	b=-0.033, p>0.05	b=0.023, p>0.05	b=0.030, p>0.05	b=-0.069, p>0.05	b=0.252, p>0.05	b=-0.016, p>0.05
**γGT_t0**	b=-0.025, p>0.05	b=0.093, p>0.05	b=-0.014, p>0.05	b=-0.059, p>0.05	b=-0.091, p>0.05	b=0.079, p>0.05	b=-0.227, p>0.05	b=0.084, p>0.05
**Total cholesterol_t0**	b=-0.032, p<0.05	b=0.031, p>0.05	b=-0.042, p>0.05	b=0.019, p>0.05	b=-0.215, p>0.05	**b=-0.271, p<0.05**	b=-0.057, p>0.05	b=-0.185, p>0.05
**HDL_t0**	b=-0.073, p>0.05	b=0.043, p>0.05	b=-0.133, p>0.05	b=0.139, p>0.05	b=0.009, p>0.05	b=0.118, p>0.05	b=-0.101, p>0.05	b=-0.054, p>0.05
**LDL_t0**	b=	b=-0.028,	b=-0.046, p>0.05	b=0.001,	b=0.127,	b=-	b=-0.137,	b=-0.141,

	0.027, p>0.05	p>0.05		p>0.05	p>0.05	0.177, p>0.05	p>0.05	p>0.05
**Calcium_t0**	b=-0.093, p>0.05	**b=-0.171, p<0.05**	**b=0.275, p<0.05**	b=-0.085, p>0.05	b=0.057, p>0.05	b=-0.057, p>0.05	b=0.151, p>0.05	b=-0.266, p>0.05
**Apo-A1_t0**	b=-0.032, p>0.05	b=-0.055, p>0.05	b=-0.039, p>0.05	b=0.073, p>0.05	b= 0.213, p>0.05	b=-0.166, p>0.05	b=-0.013, p>0.05	b=-0.129, p>0.05
**Apo-B_t0**	b=-0.030, p>0.05	b=-0.049, p>0.05	b= -0.026, p>0.05	b=-0.047, p>0.05	b= 0.049, p>0.05	b=0.069, p>0.05	b=-0.095, p>0.05	b=-0.096, p>0.05
**Lp(a)_t0**	b=-0.093, p>0.05	b=0.084, p>0.05	b=-0.130, p>0.05	b=0.098, p>0.05	b=-0.054, p>0.05	b=0.172, p>0.05	b=-0.161, p>0.05	b=0.106, p>0.05
**PTH_t0**	b=0.028, p>0.05	b=0.119, p>0.05	b=0.011, p>0.05	b=0.009, p>0.05	b=0.166, p>0.05	b=-0.078, p>0.05	b=0.158, p>0.05	b=-0.119, p>0.05
**VitaminD_t0**	b=0.024, p>0.05	b=-0.132, p>0.05	b=0.137, p>0.05	b=0.033, p>0.05	b=0.081, p>0.05	b=-0.104, p>0.05	b=-0.147, p>0.05	b=-0.204, p>0.05
**Fat percentage_t0**	b=-0.040, p>0.05	**b=0.201, p<0.05**	b=-0.095, p>0.05	b=0.069, p>0.05	b=0.120, p>0.05	b=0.020, p<0.05	b=-0.030, p>0.05	b=-0.153, p>0.05
**Fat mass_t0**	b=-0.036, p>0.05	b=0.148, p>0.05	b=-0.184, p>0.05	b=0.113, p>0.05	b=0.205, p>0.05	b=0.115, p>0.05	b=0.068, p>0.05	b=0.040, p>0.05
**Muscle mass percentage_t0**	b=-0.002, p>0.05	b=0.022, p>0.05	b=-0.128, p>0.05	b=0.055, p>0.05	b=0.180, p>0.05	b=-0.063, p>0.05	b=0.188, p>0.05	b=0.064, p>0.05
**Bone mass_t0**	b=-0.007, p>0.05	b=0.028, p>0.05	b= -0.123, p>0.05	b=0.046, p>0.05	b=0.167, p>0.05	b=0.052, p>0.05	b=0.196, p>0.05	b=0.068, p>0.05
**Free Fat mass_t0**	b=0.028, p>0.05	b=0.205, p>0.05	b=-0.127, p>0.05	b=0.055, p>0.05	b=0.179, p>0.05	b=-0.062, p>0.05	b=0.188, p>0.05	b=0.064, p>0.05
**FGF23_t0**	NA	**b= -0.632, p<0.05**	b= 0.158, p>0.05	b= -0.04, p>0.05	b= -0.027, p>0.05	b= -0.025, p>0.05	b= 0.318, p>0.05	b= 0.131, p>0.05
**Change of FGF-23**	**b=-0.632, p<0.05**	NA	**b=-0.280, p<0.05**	b=0.11, p>0.05	b=-0.099 p>0.05	b= 0.126, p>0.05	**b=0.269, p<0.05**	b= 0.042, p>0.05
**Ostepontin_t0**	b=0.158, p>0.05	**b=-0.280, p<0.05**	NA	**b=-0.780, p<0.05**	b= 0.087, p>0.05	b=-0.062, p>0.05	**b=0.643, p<0.05**	b= 0.20, p>0.05
**Change of Osteopontin**	b=-0.038, p>0.05	b= 0.114, p>0.05	**b=-0.78, p<0.05**	NA	b=-0.08, p>0.05	b= 0.031, p>0.05	b= -0.15, p>0.05	b= -0.245, p>0.05
**NGAL_t0**	b=-0.027, p>0.05	b= -0.099 p>0.05	b=0.087, p>0.05	b=-0.08, p>0.05	NA	**b=-0.890, p<0.05**	b= -0.099, p>0.05	b= 0.147, p>0.05

**Change of NGAL**	b=-0.025, p>0.05	b= 0.126, p>0.05	b=-0.062, p>0.05	b= 0.031, p>0.05	**b=-0.890, p<0.05**	NA	b= 0.188, p>0.05	b= 0.093, p>0.05
**Sclerostin t_0**	**b=0.643, p<0.05**	**b=-0.269, p<0.05**	**b=0.643, p<0.05**	b= -0.15, p>0.05	b=-0.099, p>0.05	b= 0.188, p>0.05	NA	b= -0.053, p>0.05
**Change of Sclerostin**	b=0.131, p>0.05	b= 0.042, p>0.05	b=0.20, p>0.05	b=-0.245, p>0.05	b=0.147, p>0.05	b= 0.093, p>0.05	b= -0.053, p>0.05	NA

Abbreviations: Apo-A1, apolipoprotein A1; Apo-B, apolipoprotein B; BMI, body mass index; BW, body weight; eGFR, estimated glomerular filtration rate; FGF-23, fibroblast growth factor-23; HbA1C, hemoglobin A1C; HDL, high density lipoprotein; HOMA-IR, homeostatic model assessment for insulin resistance; γGT, gamma-glutamyl transferase; LDL, low density lipoprotein; Lp(a), lipoprotein a; NGAL, neutrophil gelatinase associated lipocalin; PTH, parathormone; TMI, tri-ponderal mass index; WHR, waist-to-hip ratio; WHtR, waist-to-height ratio; Correlations of the studied variables are evaluated by the Pearson's R coefficient, and presented by b. Statistical significance was set at $p < 0.05$; Statistically significant associations are shown in bold; NA, non-applicable

Supplemental Table S2. Correlation coefficient of the assessed variables of overweight subjects at initial assessment

	FGF-23_t0	Change of FGF-23	Osteopontin_t0	Change of Osteopontin	NGAL_t0	Change of NGAL	Sclerostin_t0	Change of Sclerostin
**BW_t0**	b= -0.045, p>0.05	b=0.053, p>0.05	b= -0.030, p>0.05	b=0.106, p>0.05	b= 0.107, p>0.05	b=-0.085, p>0.05	b=0.349, p>0.05	b=-0.033, p>0.05
**BMI_z-score_t0**	b= -0.168, p>0.05	b=0.121, p>0.05	b= 0.036, p>0.05	b=-0.050, p>0.05	b= 0.209, p>0.05	**b=-0.494, p<0.05**	b= 0.260, p>0.05	b= 0.140, p>0.05
**TMI_t0**	b= -0.067, p>0.05	b= 0.008, p>0.05	b= 0.072, p>0.05	b= -0.158, p>0.05	b= 0.160, p>0.05	b=- 0.340, p>0.05	b= -0.301, p>0.05	b= -0.050, p>0.05
**Waist circumferance_t0**	b=- 0.042, p>0.05	b= 0.055, p>0.05	b= 0.183, p>0.05	b= -0.034, p>0.05	b= 0.366, p>0.05	b= -0.289, p>0.05	b= 0.472, p>0.05	**b=-0.586, p<0.05**
**WHR_t0**	b= -0.006, p>0.05	b=0.016, p>0.05	b= 0.117, p>0.05	b=-0.148, p>0.05	b= 0.198, p>0.05	b=-0.164, p>0.05	b= 0.301, p>0.05	**b=-0.613, p<0.05**
**WHtR_t0**	b= -0.036, p>0.05	b=0.049, p>0.05	b= 0.175, p>0.05	b=-0.124, p>0.05	**b=0.439, p<0.05**	b=-0.317, p>0.05	b= 0.305, p>0.05	**b=-0.575, p<0.05**
**Glucose_t0**	b=0.037, p>0.05	b=-0.056, p<0.05	**b= 0.361, p<0.05**	b=0.255, p>0.05	b= 0.054, p>0.05	b=-0.070, p>0.05	b=0.176, p>0.05	b=0.352, p>0.05
**Insulin_t0**	b=-0.059, p<0.05	b=0.025, p>0.05	b=-0.115, p>0.05	b=0.120, p>0.05	b=0.261, p>0.05	b=0.193, p>0.05	b=0.295, p<0.05	**b=0.460, p<0.05**
**HbA1c_t0**	b=-0.223, p>0.05	b=0.016, p>0.05	b=-0.022, p>0.05	b=0.026, p>0.05	b=0.299, p>0.05	b=-0.141, p>0.05	b=0.285, p>0.05	b=0.121, p>0.05
**HOMA-IR_t0**	b=-0.046, p>0.05	b=0.014, p>0.05	b=-0.015, p>0.05	b=0.024, p>0.05	**b=0.401, p<0.05**	b=-0.278, p>0.05	b=0.345, p<0.05	**b=0.557, p<0.05**
**Urea_t0**	b= -0.021, p>0.05	b=0.048, p>0.05	**b=-0.277, p<0.05**	b=0.200, p>0.05	b=-0.260, p>0.05	b=0.218, p>0.05	b=0.098, p>0.05	b=-0.166, p>0.05
**Creatinine_t0**	b=-0.018, p>0.05	b=0.014 p>0.05	b=0.110, p>0.05	b=-0.119, p>0.05	b=-0.055, p>0.05	b=0.043, p>0.05	b=0.176, p>0.05	b=-0.004, p>0.05
**eGFR_t0**	b=-0.074, p>0.05	b=0.035, p>0.05	b=-0.240, p>0.05	b=0.248, p>0.05	b=0.030, p>0.05	b=0.043, p>0.05	b=-0.059, p>0.05	b=-0.033, p>0.05
**γGT_t0**	b=-0.334, p>0.05	b=-0.010, p>0.05	b=-0.155, p>0.05	b=0.118, p>0.05	b=-0.05, p>0.05	b=-0.015, p>0.05	b=0.270, p>0.05	b=-0.224, p>0.05
**Uric acid**	b=-0.146, p>0.05	b=0.108, p>0.05	b=-0.012, p>0.05	b=-0.021, p>0.05	b=0.307, p>0.05	b=-0.352, p>0.05	b=-0.070, p>0.05	**b=0.532, p<0.05**
**Total cholesterol_t0**	b=-0.038, p>0.05	b=0.072, p>0.05	b=-0.121, p>0.05	b=0.050, p>0.05	b=-0.281, p>0.05	b=0.256, p>0.05	b=-0.278, p>0.05	b=0.175, p>0.05
**HDL_t0**	b=-0.088, p>0.05	b=0.122, p>0.05	b=-0.173, p>0.05	b=0.104, p>0.05	b=-0.168, p>0.05	b=0.204, p>0.05	b=0.006, p>0.05	b=0.016, p>0.05
**LDL_t0**	b=-0.047, p>0.05	b=0.080, p>0.05	b=-0.077, p>0.05	b=0.032, p>0.05	b=-0.240, p>0.05	b=0.197, p>0.05	b=-0.276, p>0.05	b=0.095, p>0.05
**Apo-A1_t0**	b=-0.070, p>0.05	b=0.025, p>0.05	b=-0.051, p>0.05	b=0.038, p>0.05	b=-0.124, p>0.05	b=0.157, p>0.05	b=-0.215, p>0.05	b=0.158, p>0.05
**Apo-B_t0**	b=-0.010, p>0.05	b=0.022, p>0.05	b=-0.028, p>0.05	b=0.058, p>0.05	b=-0.224, p>0.05	b=0.144, p>0.05	b=-0.359, p>0.05	b=0.158, p>0.05
**Lp(a)_t0**	b=0.0004, p>0.05	b=0.06, p>0.05	b=0.142, p>0.05	b=0.165, p>0.05	b=0.185, p>0.05	b=0.023, p>0.05	b=0.266, p>0.05	b=0.305, p>0.05

**PTH_t0**	b=-0.078, p>0.05	b=0.058, p>0.05	**b= 0.260, p<0.05**	b=-0.088, p>0.05	b=0.007, p>0.05	b=0.023, p>0.05	b=-0.033, p>0.05	b=-0.058, p>0.05
**VitaminD_t0**	b=0.130, p<0.05	b=-0.062, p>0.05	**b=0.294, p<0.05**	**b=-0.302, p<0.05**	b=-0.139, p>0.05	b=0.132, p>0.05	b=-0.309, p>0.05	b=0.334, p>0.05
**Fat percentage_t0**	b=-0.026, p<0.05	b=0.038, p<0.05	b= -0.291, p>0.05	b=0.079, p<0.05	b=0.008, p>0.05	b=0.016, p<0.05	b=-0.254, p<0.05	b=-0.380, p<0.05
**Fat mass_t0**	b=-0.063, p<0.05	b=0.086, p<0.05	b=-0.230, p>0.05	b=0.161, p<0.05	b=0.033, p>0.05	b=0.144, p<0.05	b=0.354, p<0.05	b=-0.227, p<0.05
**Muscle mass percentage_t0**	b=-0.048, p<0.05	b=0.069, p<0.05	b=-0.040, p>0.05	b=0.094, p<0.05	b= 0.034, p>0.05	b=-0.151, p<0.05	b= 0.543, p<0.05	b=0.021, p<0.05
**Bone mass_t0**	b=-0.063, p<0.05	b=0.081, p<0.05	b=-0.016, p>0.05	b=0.092, p<0.05	b= 0.090, p>0.05	b=-0.226, p<0.05	b=0.530, p<0.05	b=0.018, p<0.05
**Free Fat mass_t0**	b=-0.049, p<0.05	b=0.070, p<0.05	b= -0.039, p>0.05	b=0.094, p<0.05	b= 0.036, p>0.05	b=-0.155, p<0.05	b= 0.543, p<0.05	b=0.021, p<0.05
**FGF23_t0**	NA	**b= -0.948, p<0.05**	b=0.176, p>0.05	**b= -0.263, p<0.05**	b=-0.109, p>0.05	b=0.02, p>0.05	b= 0.318, p>0.05	b=-0.298, p>0.05
**Change of FGF-23**	**b= -0.948, p<0.05**	NA	b= -0.08, p>0.05	b=0.079, p>0.05	b=0.129, p>0.05	b=0.026, p>0.05	b=0.105, p>0.05	b=-0.204, p>0.05
**Ostepontin_t0**	b=0.176, p>0.05	b=0.08, p>0.05	NA	**b= -0.840, p<0.05**	b= 0.335, p>0.05	b=-0.229, p>0.05	b= 0.021, p>0.05	b=0.230, p>0.05
**Change of Osteopontin**	**b= -0.263, p<0.05**	b=0.079, p>0.05	**b=-0.840, p<0.05**	NA	**b=-0.398, p<0.05**	b=0.3, p>0.05	b=-0.119, p>0.05	b=-0.232, p>0.05
**NGAL_t0**	b=-0.109, p>0.05	b=0.129, p>0.05	b=0.335, p>0.05	**b= -0.398, p<0.05**	NA	**b= -0.776, p<0.05**	b= 0.170, p>0.05	b=0.110, p>0.05
**Change of NGAL**	b=0.02, p>0.05	b=0.261, p>0.05	b= -0.229, p>0.05	b=0.3, p>0.05	**b=-0.776, p<0.05**	NA	b=-0.186, p>0.05	b=-0.028, p>0.05
**Sclerostin t_0**	b=0.318, p>0.05	b=0.105, p>0.05	b= 0.021, p>0.05	b=-0.120, p>0.05	b= 0.171, p>0.05	b=-0.186, p>0.05	NA	b=-0.053, p>0.05
**Change of Sclerostin**	b=-0.298, p>0.05	b=0.205, p>0.05	b=0.23, p>0.05	b=-0.232, p>0.05	b= 0.110, p>0.05	b=-0.028, p>0.05	b=-0.053, p>0.05	NA

Abbreviations: Apo-A1, apolipoprotein A1; Apo-B, apolipoprotein B; BMI, body mass index; BW, body weight; eGFR, estimated glomerular filtration rate; FGF-23, fibroblast growth factor-23; HbA1C, hemoglobin A1C; HDL, high density lipoprotein; HOMA-IR, homeostatic model assessment for insulin resistance; γGT, gamma-glutamyl transferase; LDL, low density lipoprotein; Lp(a), lipoprotein a; NGAL, neutrophil gelatinase associated lipocalin; PTH, parathormone; TMI, tri-ponderal mass index; WHR, waist-to-hip ratio; WHtR, waist-to-height ratio; Correlations of the studied variables are evaluated by the Pearson's R coefficient, and presented by b. Statistical significance was set at $p < 0.05$; Statistically significant associations are shown in bold; NA, non-applicable

Supplemental Table S3. Correlation coefficient of the assessed variables of all subjects at initial assessment (overweight and obese)

	FGF-23_t0	Change of FGF-23	Osteoponti n_t0	Change of Osteopontin	NGAL_ t0	Change of NGAL	Sclerostin_t 0	Change of Sclerostin
**BW_t0**	b=0.066, p>0.05	b=0.019, p>0.05	b= 0.053, p>0.05	b=-0.094, p>0.05	b=0.163, p>0.05	b=0.092, p>0.05	b=0.076, p>0.05	b=0.145, p>0.05
**BMI_z-score_t0**	b=0.056, p>0.05	b=0.007, p>0.05	b=-0.353, p>0.05	b=-0.042, p>0.05	b=0.091, p>0.05	b=0.065, p>0.05	b=0.270, p>0.05	b=0.197, p>0.05
**TMI_t0**	b= 0.005, p>0.05	b=0.060, p>0.05	b=-0.043, p>0.05	b=-0.067, p>0.05	b= 0.174, p>0.05	b=0.28, p>0.05	b= 0.171, p>0.05	b=0.199, p>0.05
**Waist circumferance _t0**	b= 0.107, p>0.05	b=-0.033, p>0.05	b= 0.173, p>0.05	b=-0.112, p>0.05	b= 0.237, p>0.05	b=-0.033, p>0.05	b= 0.014, p>0.05	b=0.244, p>0.05
**WHR_t0**	b= 0.238, p>0.05	b=-0.238, p>0.05	b= -0.047, p>0.05	b=0.025, p>0.05	b= 0.103, p>0.05	b=-0.050, p>0.05	b= -0.186, p>0.05	b=-0.087, p>0.05
**WHtR_t0**	b= 0.094, p>0.05	b=-0.041, p>0.05	b= 0.174, p>0.05	b=-0.243, p>0.05	b= 0.247, p>0.05	b=-0.133, p>0.05	b= 0,032, p>0.05	b=0.296, p>0.05
**Glucose_t0**	b=0.142, p>0.05	b=-0.158, p>0.05	**b=0.366, p<0.05**	**b=-0.320, p<0.05**	b=- 0.077, p>0.05	b=0.109, p>0.05	b= 0.163, p>0.05	**b=0.315, p<0.05**
**Insulin**	b=-0.008, p>0.05	b=0.009, p>0.05	b=0.069, p>0.05	b=-0.153, p>0.05	b=0.093, p>0.05	b=0.009, p>0.05	b=0.081, p>0.05	b=0.285, p>0.05
**HbA1c**	b=-0.070, p>0.05	b=0.107, p>0.05	b=0.202, p>0.05	b=-0.172, p>0.05	b=0.267, p>0.05	**b=-0.304, p<0.05**	b=0.009, p>0.05	b=0.166, p>0.05
**HOMA-IR**	b=0.006, p>0.05	b=-0.002, p>0.05	b=0.104, p>0.05	b=-0.180, p>0.05	b=0.067, p>0.05	b=0.035, p>0.05	b=0.093, p>0.05	**b=0.300, p<0.05**
**Urea_t0**	b=-0.173, p>0.05	b=0.172, p>0.05	b=-0.219, p>0.05	b=0.137, p>0.05	b=- 0.226, p>0.05	b=0.161, p>0.05	**b=-0.324, p<0.05**	b=-0.058, p>0.05
**Creatinine_t0**	b=0.017, p>0.05	b=0.100, p>0.05	b=0.113, p>0.05	b=-0.050, p>0.05	b=- 0.133, p>0.05	b=0.124, p>0.05	b=-0.147, p>0.05	b=0.011, p>0.05
**eGFR_t0**	b=0.059, p>0.05	b=-0.177, p>0.05	b=-0.120, p>0.05	b=0.074, p>0.05	b=0.144, p>0.05	b=-0.135, p>0.05	b=0.265, p>0.05	b=-0.052, p>0.05
**γGT_t0**	b=-0.156, p>0.05	b=0.161, p>0.05	b=0.038, p>0.05	b=-0.065, p>0.05	b=-0.02, p>0.05	b=0.082, p>0.05	**b=-0.329, p<0.05**	b=0.074, p>0.05
**Total cholesterol_t0**	b=0.025, p>0.05	b=-0.096, p>0.05	b=0.063, p>0.05	b=-0.141, p>0.05	b=0.142, p>0.05	b=-0.218, p>0.05	b=-0.005, p>0.05	b=-0.126, p>0.05
**HDL_t0**	b=-0.123, p>0.05	b=0.160, p>0.05	b=-0.126, p>0.05	b=0.215, p>0.05	b=- 0.157, p>0.05	b=0.229, p>0.05	b=-0.100, p>0.05	b=-0.016, p>0.05
**LDL_t0**	b=0.020, p>0.05	b=0.069, p>0.05	b=-0.001, p>0.05	b=-0.158, p>0.05	b=0.034, p>0.05	b=-0.050, p>0.05	b=-0.130, p>0.05	b=-0.094, p>0.05
**ApoA1_t0**	b=-0.007, p>0.05	b=-0.118, p>0.05	b=-0.079, p>0.05	b=0.013, p>0.05	b=0.073, p>0.05	b=-0.156, p>0.05	b=-0.116, p>0.05	b=0.093, p>0.05
**ApoB_t0**	b=0.028, p>0.05	b=0.035, p>0.05	b=-0.002, p>0.05	b=-0.216, p>0.05	b=0.029, p>0.05	b=0.095, p>0.05	b=-0.153, p>0.05	b=0.027, p>0.05

**Lp(a)_t0**	b=-0.195, p>0.05	b=0.242, p>0.05	b=-0.006, p>0.05	b=-0.081, p>0.05	b=-0.010, p>0.05	b=0.089, p>0.05	b=-0.223, p>0.05	b=0.098, p>0.05
**Fat percentage_t0**	b=-0.264, p>0.05	**b=0.313, p<0.05**	b=-0.259, p>0.05	b=0.271, p>0.05	b=0.038, p>0.05	b=0.100, p>0.05	b=-0.004, p>0.05	b=-0.084, p>0.05
**Fat mass_t0**	b=-0.068, p>0.05	b=0.123, p>0.05	b=-0.224, p>0.05	b=0.169, p>0.05	b=0.166, p>0.05	b=0.018, p>0.05	b=-0.115, p>0.05	b=0.003, p>0.05
**Muscle mass percentage_t0**	b=0.200, p>0.05	b=0.136, p>0.05	b=-0.049, p>0.05	b=0.014, p>0.05	b=0.203, p>0.05	b=0.084, p>0.05	b=0.243, p>0.05	b=0.050, p>0.05
**Bone mass_t0**	b=0.196, p>0.05	b=-0.135, p>0.05	b=-0.052, p>0.05	b=-0.010, p>0.05	b=0.205, p>0.05	b=-0.092, p>0.05	b=0.249, p>0.05	b=-0.052, p>0.05
**Free Fat mass_t0**	b=0.199, p>0.05	b=-0.136, p>0.05	b=-0.048, p>0.05	b=-0.014, p>0.05	b=0.203, p>0.05	b=0.084, p>0.05	b=0.243, p>0.05	b=0.050, p>0.05
**FGF23_t0**	NA	**b=-0.915, p<0.05**	**b=0.574, p<0.05**	b=-0.077, p>0.05	b=-0.004, p>0.05	b=-0.086, p>0.05	**b=0.443, p<0.05**	b=-0.103, p>0.05
**Change of FGF-23**	**b=-0.946, p<0.05**	NA	b=0.055, p>0.05	b=0.124, p>0.05	b=0.050, p>0.05	b=0.104, p>0.05	**b=-0.344, p<0.05**	b=0.024, p>0.05
**Ostepontin_t0**	**b=0.574, p<0.05**	**b=-0.534, p<0.05**	NA	**b=-0.839, p<0.05**	b=-0.192, p>0.05	b=-0.109, p>0.05	b=0.250, p>0.05	**b=0.299, p<0.05**
**Change of Osteopontin**	**b=-0.489, p<0.05**	**b=0.442, p<0.05**	**b=-0.839, p<0.05**	NA	**b=-0.332, p<0.05**	b=0.146, p>0.05	b=-0.140, p>0.05	**b=-0.352, p<0.05**
**NGAL_t0**	b=0.004, p>0.05	b=-0.088, p>0.05	b=0.192, p>0.05	**b=-0.332, p<0.05**	NA	**b=-0.882, p<0.05**	b=-0.112, p>0.05	b=0.236, p>0.05
**Change of NGAL**	b=-0.039, p>0.05	b=0.146, p>0.05	b=-0.109, p>0.05	b=0.146, p>0.05	**b=0.547 , p<0.05**	NA	b=0.099, p>0.05	b=-0.146, p>0.05
**Sclerostin t_0**	**b=0.443, p<0.05**	**b=-0.344, p<0.05**	b=-0.156, p>0.05	b=-0.140, p>0.05	b=-0.112, p>0.05	b=0.099, p>0.05	NA	b=-0.071, p>0.05
**Change of Sclerostin**	**b=0.385, p<0.05**	**b=-0.357, p<0.05**	**b=0.299, p<0.05**	**b=-0.352, p<0.05**	b=0.236, p>0.05	b=-0.146, p>0.05	b=-0.071, p>0.05	NA

Abbreviations: Apo-A1, apolipoprotein A1; Apo-B, apolipoprotein B; BMI, body mass index; BW, body weight; eGFR, estimated glomerular filtration rate; FGF-23, fibroblast growth factor-23; HbA1C, hemoglobin A1C; HDL, high density lipoprotein; HOMA-IR, homeostatic model assessment for insulin resistance; γGT, gamma-glutamyl transferase; LDL, low density lipoprotein; Lp(a), lipoprotein a; NGAL, neutrophil gelatinase associated lipocalin; TMI, tri-ponderal mass index; WHR, waist-to-hip ratio; WHtR, waist-to-height ratio; Correlations of the studied variables are evaluated by the Pearson's R coefficient, and presented by b. Statistical significance was set at $p < 0.05$; Statistically significant associations are shown in bold; NA, non-applicable
